# Genetic evaluation of encorafenib-associated kinases *RAF1* and *MAPK8* in pancreatic cancer: Exploratory mediation by circulating immune traits

**DOI:** 10.1016/j.tranon.2026.103040

**Published:** 2026-09-22

**Authors:** Fen Lin, Wenwen Tu, Ronggui Lin, Han Shi, Shiyun Lu, Zongqi Weng

**Affiliations:** a Department of Cardiology Nursing, Fujian Medical University Union Hospital, Fuzhou, Fujian, 350001, China; b Department of General Practice, Fujian Medical University Union Hospital, Fuzhou, Fujian, 350001, China; c Department of General Surgery, Fujian Medical University Union Hospital, Fuzhou, Fujian, 350001, China; d Key Laboratory of Nanomedical Technology (Education Department of Fujian Province), Department of Pharmaceutical Analysis, School of Pharmacy, Fujian Medical University, Fuzhou, Fujian, China; e Shengli Clinical Medical College, Fujian Medical University, Fuzhou, Fujian, China; f Department of Gastroenterology, Fuzhou University Affiliated Provincial Hospital, Fuzhou, Fujian, China

**Keywords:** Pancreatic cancer, Mendelian randomization, regulatory T cells, *RAF1*, *MAPK8*

## Abstract

**Objectives:**

Pancreatic cancer (PC) is a highly lethal malignancy, and identifying actionable therapeutic targets remains crucial. *RAF1* and *MAPK8* are kinases linked to the in vitro pharmacological profile of encorafenib. This study investigated the associations of genetically proxied *RAF1* and *MAPK8* expression with PC risk and explored the potential mediating role of circulating immune traits.

**Methods:**

We implemented a two-sample, two-step Mendelian randomization framework integrating expression quantitative trait locus data for *RAF1* and *MAPK8* with genome-wide association study data for PC and 731 immune cell phenotypes. Causal estimates were primarily derived using inverse-variance weighting, and mediation analysis assessed possible immune cell-dependent effects. A phenome-wide association study (PheWAS) evaluated potential genetic pleiotropy.

**Results:**

Genetically proxied *RAF1* expression (OR = 0.748, 95% CI: 0.633–0.882, P = 0.001) and *MAPK8* expression (OR = 0.829, 95% CI: 0.740–0.929, *P* = 0.001) were significantly associated with a lower risk of PC. An activated and resting CD4+ regulatory T-cell-related phenotype showed a possible partial mediating effect. The estimated mediation proportions were 16.202% for *MAPK8* and 10.607% for *RAF1*, suggesting that Treg-related immune variation may account for part of these associations. In the PheWAS analysis, no association reached the prespecified significance threshold, and no broad genetic pleiotropic signal was detected.

**Conclusions:**

This study provides genetic evidence linking *RAF1* and *MAPK8* to PC risk. A circulating regulatory T-cell-related phenotype showed exploratory evidence of a possible partial contribution. These findings support further functional and pharmacological investigation of *RAF1*- and *MAPK8*-related pathways in pancreatic cancer.

## Highlights

• Genetically proxied RAF1 and MAPK8 expression was associated with pancreatic cancer risk.
• A circulating CD4+ Treg-related phenotype showed a possible partial indirect contribution.
• PheWAS detected no broad genetic pleiotropic signals at the prespecified threshold.
• The findings support further study of RAF1- and MAPK8-related pathways in pancreatic cancer.

## Graphical abstract

**Figure:**
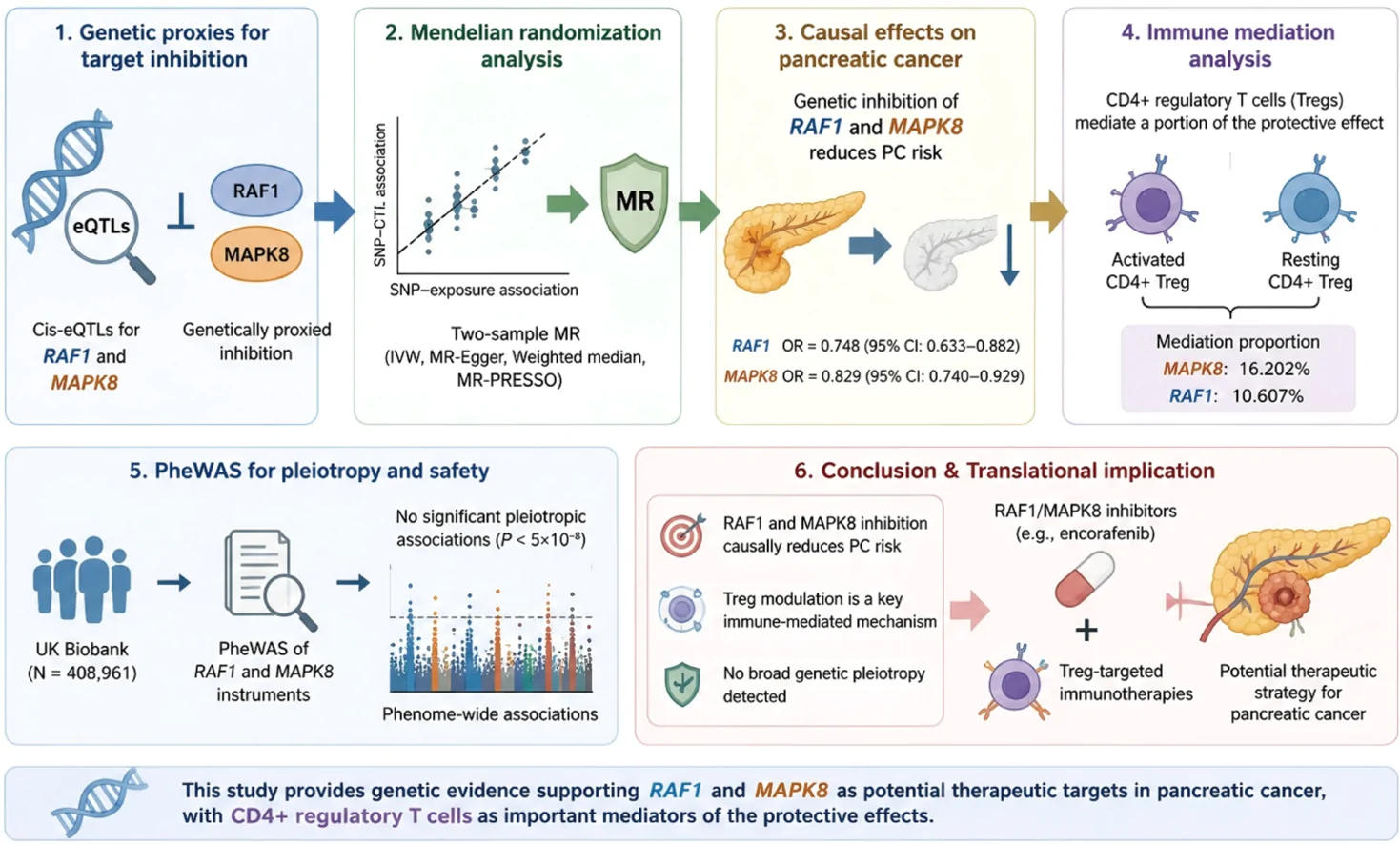

## Introduction

Pancreatic cancer (PC) remains one of the most lethal malignancies worldwide, with a five-year survival rate stubbornly below 10% [[1], [2], [3]]. Its insidious onset and rapid progression often lead to late-stage diagnosis, severely limiting the efficacy of conventional therapies such as surgery and chemotherapy [2,4]. The projected doubling of PC-related deaths by 2060 underscores the urgent, unmet need for novel therapeutic strategies [5,6].

In this context, modulating the immunosuppressive tumor microenvironment (TME) has emerged as a promising frontier [7]. PC is characterized by a profoundly immunosuppressive TME, where mechanisms such as immune checkpoint activation and infiltration of immunosuppressive cells, including regulatory T cells (Tregs), facilitate immune evasion and promote tumor progression [[8], [9], [10], [11]]. Consequently, strategies to overcome this immune barrier are of paramount importance.

The mitogen-activated protein kinase (MAPK) signaling pathway is a cornerstone of PC pathogenesis, frequently activated by oncogenic KRAS mutations and driving uncontrolled proliferation and survival [12]. Beyond its cell-autonomous oncogenic role, the MAPK pathway critically shapes the immune landscape of the TME, influencing responses to both chemotherapy and immunotherapy [13]. This dual function makes it an attractive therapeutic target. Notably, MAPK pathway inhibitors, such as RAF kinase inhibitors, have revolutionized the treatment of cancers such as BRAF-mutant melanoma, where they not only directly inhibit tumor growth but also synergize with immunotherapy by modulating immune responses [14]. Beyond direct RAF inhibition, pharmacological targeting of upstream regulators of RAS–MAPK signaling, such as SHP2, has also emerged as a potential anticancer strategy, reflecting the broader therapeutic relevance of modulating this signaling network [15]. Similar principles of targeting MAPK to overcome resistance are being explored for other malignancies, such as EGFR-mutant non-small cell lung cancer [16]. These successes raise a pivotal, yet unexplored, question: Could therapeutic targeting of the MAPK pathway be harnessed in PC by leveraging its immunomodulatory effects?

Encorafenib, a selective RAF kinase inhibitor approved for BRAF-mutant melanoma, may represent a potential candidate for further investigation in PC [17]. Pharmacological evidence indicates that encorafenib can inhibit CRAF, encoded by *RAF1*, and bind JNK1, encoded by *MAPK8*, in vitro, linking these kinases to its broader pharmacological profile. Preliminary evidence suggests that encorafenib may affect PC through the modulation of MAPK-related signaling [18]. However, its mechanism of action in PC remains unclear, particularly whether immune regulation, including Treg-related responses, contributes to its potential effects. Clarifying these relationships may help define the biological relevance of *RAF1*- and *MAPK8*-related pathways and inform further experimental evaluation in PC.

To address this question, we employed Mendelian randomization (MR), an established genetic epidemiological method that uses genetic variants as instrumental variables to assess potential causal relationships while reducing the influence of confounding and reverse causation [19,20]. We hypothesized that genetically proxied perturbations of the encorafenib-associated kinases *RAF1* and *MAPK8* may be associated with a reduced risk of PC and that circulating immune cell phenotypes, including Treg-related traits, may partially mediate these associations. Therefore, this study aimed to systematically investigate the relationships among *RAF1* and *MAPK8*, circulating immune cell subtypes, and PC risk. Our findings were intended to provide gene-level evidence on the associations of *RAF1* and *MAPK8* with PC risk and to explore the possible contribution of circulating immune traits.

## Materials and methods

### Study design

In this eQTL-based study, a two-sample, two-step MR framework was used to examine the associations of genetically proxied *RAF1* and *MAPK8* expression with circulating immune phenotypes and PC risk (Fig. 1). To ensure the validity of the IVs and the credibility of the study results, the following core assumptions were followed. First, single nucleotide polymorphisms (SNPs) were significantly associated with the expression of *RAF1* or *MAPK8* (*P* < 1 × 10^-5^, F statistic > 10), indicating adequate instrument strength. Second, SNPs were independent of potential confounders, thereby minimizing the risk of bias. Furthermore, SNPs affect PC through genetically proxied *RAF1*/*MAPK8* expression rather than directly, as verified by the MR‒Egger regression model, with an intercept *P* > 0.05, indicating no significant pleiotropy [21]. Additionally, adherence to the Strengthening the Reporting of Observational Studies in Epidemiology using Mendelian Randomization (STROBE-MR) reporting guidelines was maintained to ensure transparency and standardization [22]. Because this study used cis-eQTL instruments to evaluate genetically proxied gene expression, a less stringent threshold than genome-wide significance thresholds used for complex trait GWAS was applied to obtain sufficient expression-associated instruments. Additional procedures, including cis-region restriction, linkage disequilibrium pruning, PhenoScanner-based filtering, and sensitivity analyses, were performed to minimize potential bias.

**Fig. 1:**
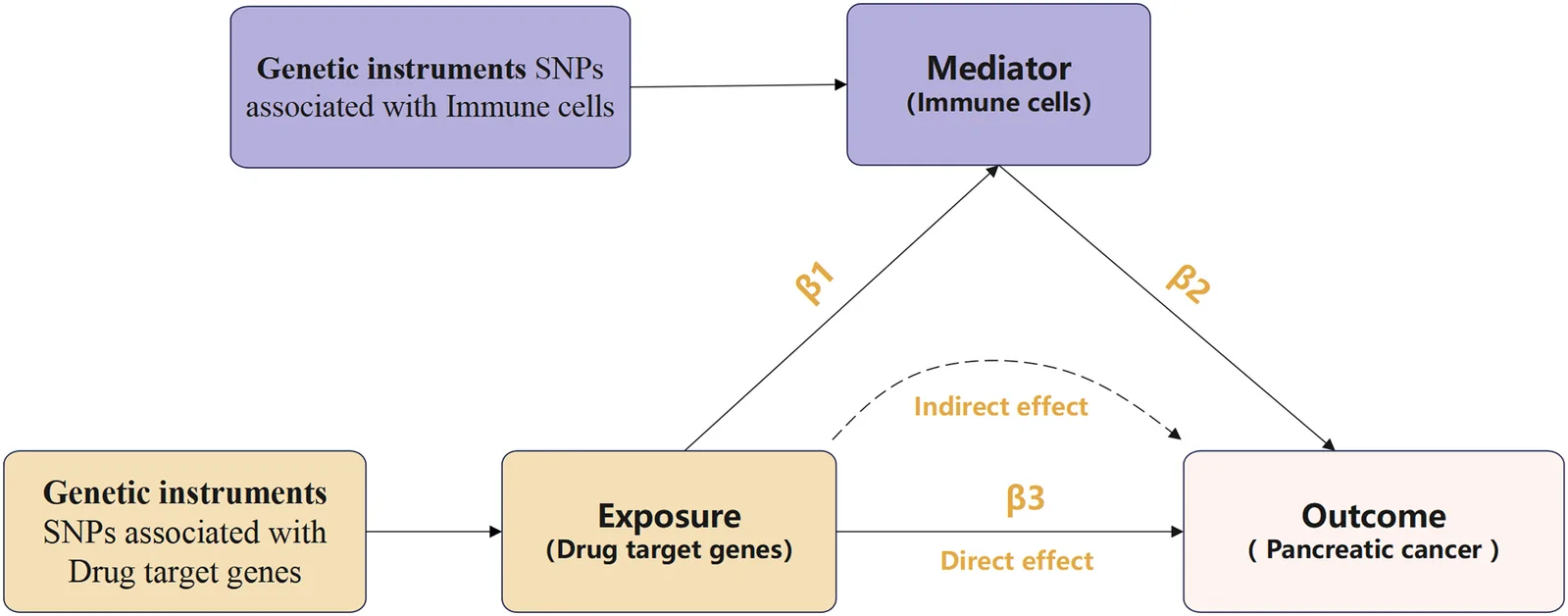
Mendelian randomization analysis of the immune cell-mediated effect of genetic expression of *RAF1*/*MAPK8* on pancreatic cancer.

Within this framework, we first estimated the associations of genetically proxied *RAF1* and *MAPK8* expression with PC risk and subsequently explored candidate circulating immune pathways using two-step MR analysis.

### Data sources

Summary-level genetic data were obtained from the IEU OpenGWAS Project (https://gwas.mrcieu.ac.uk/; accessed 13 October 2024), including eQTL data for nine candidate genes (CCND1, *RAF1*, STK36, MAPK9, *MAPK8*, LIMK2, MAP2K4, LIMK1, and BRAF) and GWAS datasets for 731 immune cell phenotypes, pancreatic cancer, and three comparison cancer outcomes. All the data were fully anonymized without access to individual identifiers. The original studies obtained participant consent and ethics approval. In accordance with institutional guidelines, secondary analysis of public aggregate data requires no additional ethics review. The data sources were restricted to European populations (Table 1).

**Table 1:** eQTLs and GWAS datasets utilized in the MR analysis.

**Data source**	**Phenotype**	**Sample size**	**Cases**	**Population**	**Adjustment**
eqtl-a-ENSG00000110092	CCND1	31684	-	European	Males and Females
eqtl-a-ENSG00000132155	*RAF1*	31684	-	European	Males and Females
eqtl-a-ENSG00000163482	STK36	31684	-	European	Males and Females
eqtl-a-ENSG00000050748	MAPK9	31430	-	European	Males and Females
eqtl-a-ENSG00000107643	*MAPK8*	31256	-	European	Males and Females
eqtl-a-ENSG00000182541	LIMK2	30721	-	European	Males and Females
eqtl-a-ENSG00000065559	MAP2K4	14263	-	European	Males and Females
eqtl-a-ENSG00000106683	LIMK1	14263	-	European	Males and Females
eqtl-a-ENSG00000157764	BRAF	14263	-	European	Males and Females
IEU Open GWAS project	Immune cells	-	-	European	-
finn-b-C3_NSCLC_ADENO	Non-small cell lung cancer, adenocarcinoma	218792	571	European	Males and Females
finn-b-C3_THYROID_GLAND	Malignant neoplasm of the thyroid gland	218792	989	European	Males and Females
finn-b-CD2_NONHODGKIN_NAS	Other and unspecified types of non-Hodgkin lymphoma	218792	533	European	Males and Females
ebi-a-GCST90018893	Pancreatic cancer	476245	1196	European	-

eQTLs, expression quantitative trait loci; GWAS, genome-wide association study; MR, Mendelian randomization.

### Selection of IVs

#### eQTL instruments for candidate genes

To ensure biological relevance, the IVs were restricted to the cis-acting regions of the target gene within a 100 kb range. Relevant SNPs were extracted from the GWAS summary data of the outcome variables (PC and immune cells). Palindromic SNPs and those with a minor allele frequency (MAF) > 0.42 were excluded. Outlier SNPs were further removed using Mendelian randomization pleiotropy residual sum and outlier (MR-PRESSO). Additionally, linkage disequilibrium (LD) was controlled by setting the LD coefficient (r²) to 0.3 and the LD region width to 100 kb, with an MAF > 0.01, to ensure independence between SNPs and eliminate the influence of LD on the results. SNPs associated with confounding factors or outcomes were excluded using the PhenoScanner tool (http://www.phenoscanner.medschl.cam.ac.uk/).

#### eQTL IVs for immune cells

To ensure the independence of the SNPs and minimize the influence of LD on the results, a more stringent threshold was applied. The LD threshold was set at r² < 0.001, with a window size of 10,000 kb. The MAF threshold was set to > 0.01. The PhenoScanner tool was used to exclude SNPs associated with potential confounders or outcome variables (PC).

### Statistical analysis

Statistical analyses were conducted using the TwoSampleMR package in R (version 4.1.0). A nominal significance threshold of P < 0.05 was used for exploratory screening analyses to identify candidate associations. Given the number of candidate genes and circulating immune phenotypes evaluated, nominally significant secondary associations were interpreted cautiously and were considered hypothesis-generating rather than confirmatory.

#### Causal relationships analysis

To investigate the causal relationships between the exposure, defined as genetically proxied *RAF1* or *MAPK8* expression and the outcome (PC), multiple regression models, including MR‒Egger regression, inverse-variance weighting (IVW), a weighted median estimator (WME), the weighted mode, and the simple mode, were employed in this study. For all regression models, eQTLs were utilized as IVs, and two-sample MR analysis was applied to evaluate the association of genetically proxied *RAF1* or *MAPK8* expression with PC risk. The IVW method served as the primary analytical approach, whereas the other methods were used for complementary analysis.

When the number of SNPs (N_snp) was ≤ 3, the Wald ratio was used to assess the effect of individual SNPs on the outcome, whereas the fixed-effects IVW method was applied in other cases. For N_snp > 3, random-effects IVW was employed. In cases where horizontal pleiotropy of SNPs was present, the results of MR‒Egger regression were used for reference.

#### Sensitivity analysis

The pleiotropy test was conducted using MR‒Egger regression. A nonsignificant MR-Egger intercept test (*P* > 0.05) was considered to indicate no evidence of directional horizontal pleiotropy.

Heterogeneity was assessed using Cochran's Q test, with *P <* 0.05 indicating the presence of heterogeneity. An I² > 50% suggested significant heterogeneity. Furthermore, a leave-one-out sensitivity analysis was performed by sequentially removing each SNP to evaluate the impact of each SNP on the final result.

#### Mediation effect analysis

Given the potential role of immune cells in PC, a two-step MR approach was used to explore whether circulating immune phenotypes represented candidate indirect pathways in the associations between genetically proxied *RAF1*/*MAPK8* expression and PC risk. First, the causal effect of *RAF1* or *MAPK8* expression on immune cell phenotypes was estimated using target gene expression-associated SNPs (β_1_). Second, the causal effect of immune cell phenotypes on PC was estimated using immune cell-associated SNPs (β_2_). The total effect of genetically proxied *RAF1* or *MAPK8* expression on PC was denoted as β_3_. The mediation proportion was calculated as follows:

$$E\left(\%\right)=\left(\beta_{1}\times \beta_{2}\right)/\beta_{3}$$

where β₃ represents the total effect of genetically proxied *RAF1* or *MAPK8* expression on PC. The mediation proportion was used to estimate the relative contribution of each circulating immune phenotype to the total association between genetically proxied *RAF1* or *MAPK8* expression and PC risk. Positive mediation proportions indicate indirect effects operating in the same direction as the total effect, whereas negative values indicate opposite directional effects and may reflect suppression rather than conventional mediation. Treg-related traits were of particular biological interest given their established relevance to immune regulation in cancer; however, no single circulating immune phenotype was designated as the sole mediator before the exploratory screening.

### PheWAS analysis

To evaluate broad genetic phenotype associations and potential pleiotropic signals related to *RAF1* and *MAPK8*, we performed a PheWAS via the AstraZeneca PheWAS portal, which provides gene–phenotype associations across 17,361 binary and 1,419 quantitative phenotypes. Genetic instruments for *MAPK8* and *RAF1* were tested across these phenotypes using logistic regression for binary phenotypes and linear regression for quantitative phenotypes, with a genome-wide significance threshold of *P* < 5 × 10^-8^. Manhattan plots were used to visualize the PheWAS results.

### Data availability statement

All data used in this study are available in a public repository. The code involved in the data analysis processes can be obtained by contacting the corresponding author.

## Results

### Results of SNP selection

We identified 60 SNPs associated with PC (Supplementary Table S1) and 40,310 SNPs associated with immune cells (Supplementary Table S2) from eQTL data for *RAF1* and *MAPK8*. Additionally, 10,936 PC-related SNPs were obtained from immune cell GWAS data (Supplementary Table S3), and 95 SNPs associated with the three comparison cancer outcomes (non-small cell lung cancer, malignant neoplasm of the thyroid gland, and non-Hodgkin lymphoma) were identified in the same eQTL data (Supplementary Table S4).

### Associations of genetically proxied RAF1 and MAPK8 expression with pancreatic cancer risk

IVW analysis demonstrated that expression of both *MAPK8* (odds ratio [OR] = 0.829, 95% confidence interval [CI]: 0.740–0.929, *P* = 0.001) and *RAF1* (OR = 0.748, 95% CI: 0.633–0.882, *P* = 0.001) was significantly associated with a reduced risk of PC (Table 2). No significant heterogeneity for either expression was found in any sensitivity or pleiotropy analysis (Supplementary Figs. S1 and S2). Among the additional candidate genes evaluated, genetically proxied MAP2K4 expression showed a nominal IVW association with PC risk (OR = 1.533, 95% CI: 1.099–2.137, *P* = 0.012), whereas the remaining candidate genes showed no nominal evidence of association. Given the exploratory nature of these secondary candidate-gene analyses, the MAP2K4 finding was considered hypothesis-generating rather than confirmatory.

**Table 2:** Mendelian randomization estimates for the associations of genetically proxied candidate-gene expression with pancreatic cancer risk.

**Exposure**	**Outcome**	**Method**	**Nsnp**	**MR**		**Heterogeneity**			**Horizontal pleiotropy**			**MR-PRESSO**
				**OR (95% CI)**	***P* value**	**I^2^ (%)**	**Cochran's Q**	***P* value**	**Egger intercept**	**SE**	***P* value**	***P* value**
*RAF1* expression	PC	Inverse variance weighted	19	0.748(0.633-0.882)	0.001	0	12.759	0.806				0.877
		MR–Egger	19	0.724(0.437-1.199)	0.226	0	12.742	0.753	-0.005	0.039	0.897	
		Weighted median	19	0.762(0.612-0.950)	0.016							
		Simple mode	19	0.751(0.557-1.014)	0.078							
		Weighted mode	19	0.756(0.609-0.938)	0.020							
*MAPK8* expression	PC	Inverse variance weighted	41	0.829(0.740-0.929)	0.001	10	44.341	0.294				0.365
		MR–Egger	41	0.931(0.720-1.202)	0.585	10	43.259	0.294	0.026	0.026	0.329	
		Weighted median	41	0.847(0.714-1.005)	0.057							
		Simple mode	41	0.892(0.659-1.207)	0.463							
		Weighted mode	41	0.892(0.685-1.162)	0.402							
MAP2K4 expression	PC	Inverse variance weighted	6	1.533(1.099-2.137)	0.012	0	1.775	0.879				0.510
		MR–Egger	6	1.146(0.582-2.257)	0.714	0	0.843	0.933	-0.045	0.047	0.389	
		Weighted median	6	1.391(0.936-2.068)	0.102							
		Simple mode	6	1.388(0.786-2.452)	0.310							
		Weighted mode	6	1.338(0.816-2.194)	0.300							
LIMK2 expression	PC	Inverse variance weighted	4	0.616(0.370-1.027)	0.063	0	1.391	0.708				0.793
		MR–Egger	4	3.176(0.150-67.366)	0.536	0	0.252	0.882	0.147	0.138	0.398	
		Weighted median	4	0.563(0.301-1.053)	0.072							
		Simple mode	4	0.549(0.242-1.246)	0.247							
		Weighted mode	4	0.557(0.272-1.141)	0.208							
STK36 expression	PC	Inverse variance weighted	7	1.059(0.897-1.251)	0.496	0	2.208	0.900				0.910
		MR–Egger	7	0.744(0.406-1.363)	0.383	0	0.796	0.977	-0.095	0.080	0.288	
		Weighted median	7	1.096(0.897-1.339)	0.368							
		Simple mode	7	1.105(0.867-1.408)	0.452							
		Weighted mode	7	1.107(0.886-1.382)	0.407							
BRAF expression	PC	Inverse variance weighted	2	0.983(0.418-2.312)	0.969	0	0.405	0.525				
CCND1 expression	PC	Inverse variance weighted	16	1.128(0.945-1.347)	0.182	13	17.272	0.303				0.467
		MR–Egger	16	1.447(0.912-2.298)	0.139	11	15.803	0.326	0.046	0.040	0.273	
		Weighted median	16	1.138(0.908-1.425)	0.262							
		Simple mode	16	1.129(0.797-1.600)	0.505							
		Weighted mode	16	1.148(0.896-1.471)	0.291							
LIMK1 expression	PC	Inverse variance weighted	32	1.025(0.948-1.108)	0.537	0	18.120	0.968				0.922
		MR–Egger	32	0.897(0.771-1.044)	0.172	0	14.124	0.994	-0.049	0.024	0.055	
		Weighted median	32	0.981(0.880-1.094)	0.735							
		Simple mode	32	1.012(0.848-1.207)	0.898							
		Weighted mode	32	0.978(0.893-1.073)	0.645							
MAPK9 expression	PC	Inverse variance weighted	60	0.929(0.850-1.015)	0.102	0	56.097	0.583				0.527
		MR–Egger	60	0.904(0.750-1.091)	0.297	0	55.996	0.550	-0.005	0.016	0.752	
		Weighted median	60	0.934(0.820-1.064)	0.304							
		Simple mode	60	0.968(0.763-1.227)	0.786							
		Weighted mode	60	0.920(0.787-1.075)	0.297							

MR-PRESSO analysis was not feasible when N_snp ≤ 3.

MR, Mendelian randomization; PC, pancreatic cancer; OR, odds ratio; CI, confidence interval; SE, standard error; MR-PRESSO, Mendelian randomization pleiotropy residual sum and outlier.

### Exploratory associations of RAF1 and MAPK8 expression with circulating immune phenotypes

At the nominal significance threshold of *P* < 0.05, exploratory IVW analysis identified candidate associations between genetically proxied *MAPK8* expression and 196 circulating immune phenotypes (Supplementary Table S5). Of these phenotypes, 76 showed inverse associations and 120 showed positive associations. HLA-DR on HLA-DR+ T cells had the lowest OR (OR = 0.786, 95% CI: 0.734–0.842, *P* < 0.001), whereas CD45RA+ CD28− CD8br AC had the highest OR (OR = 22,428.702, 95% CI: 1.127–446,420,959.281, *P* = 0.047).

Similarly, exploratory analysis identified nominal associations between genetically proxied *RAF1* expression and 207 circulating immune phenotypes, including 140 inverse associations and 67 positive associations. CD45 on CD33− HLA-DR− cells had the lowest OR (OR = 0.669, 95% CI: 0.568–0.789, *P* < 0.001), while CD45RA on naive CD8br cells had the highest OR (OR = 1.371, 95% CI: 1.215–1.548, *P* < 0.001).

Sensitivity analyses for these representative associations are presented in Supplementary Fig. S3–S6. These results were used to identify candidate circulating immune phenotypes for the subsequent two-step MR analyses.

### Exploratory associations between circulating immune phenotypes and pancreatic cancer risk

At the nominal significance threshold of *P* < 0.05, IVW analysis identified associations between 35 circulating immune phenotypes and pancreatic cancer risk (Table 3). Among these phenotypes, 18 showed inverse associations and 17 showed positive associations. CD86+ plasmacytoid DC AC had the lowest OR (OR = 0.768, 95% CI: 0.634–0.931, *P* = 0.007), whereas TD CD4+ %T cells had the highest OR (OR = 1.398, 95% CI: 1.106–1.766, *P* = 0.005). Sensitivity analyses for these representative phenotypes are presented in Supplementary Fig. S7 and S8.

**Table 3:** MR analysis results for the causal relationship between immune cells and PC.

**Exposure**	**Outcome**	**Type**	**Panel**	**Nsnp**	**MR**		**Heterogeneity**			**Horizontal pleiotropy**			**MR-PRESSO**
					**OR (95% CI)**	***P* value**	**I^2^ (%)**	**Cochran's Q**	***P* value**	**Egger intercept**	**SE**	***P* value**	***P* value**
CD86+ plasmacytoid DC AC	Pancreatic cancer	Absolute count	cDC	10	0.768(0.634-0.931)	0.007	0	3.997	0.912	-0.055	0.069	0.448	0.681
CD39+ activated Treg AC	Pancreatic cancer	Absolute count	Treg	20	1.113(1.004-1.233)	0.041	0	17.945	0.526	0.014	0.024	0.560	0.229
Activated & resting Treg % CD4 Treg	Pancreatic cancer	Relative count	Treg	14	0.850(0.724-0.999)	0.049	0	8.359	0.819	-0.029	0.039	0.474	0.888
TD CD4+ %T cell	Pancreatic cancer	Relative count	Maturation stages of T cell	11	1.398(1.106-1.766)	0.005	0	9.785	0.460	0.096	0.068	0.191	0.123
CM DN (CD4-CD8-) %DN	Pancreatic cancer	Relative count	Maturation stages of T cell	9	0.787(0.622-0.996)	0.047	35	12.271	0.140	-0.047	0.049	0.368	0.172
CD14+ CD16- monocyte %monocyte	Pancreatic cancer	Relative count	Monocyte	17	0.792(0.678-0.925)	0.003	7	17.216	0.372	-0.078	0.029	0.017	0.331
CD16+ monocyte %monocyte	Pancreatic cancer	Relative count	Monocyte	19	1.206(1.045-1.393)	0.010	4	18.807	0.404	0.063	0.028	0.037	0.405
CD8br %T cell	Pancreatic cancer	Relative count	TBNK	16	1.224(1.003-1.492)	0.046	49	29.254	0.015	0.020	0.056	0.730	0.036
Leukocyte AC	Pancreatic cancer	Absolute count	TBNK	7	1.238(1.027-1.492)	0.025	0	5.595	0.470	-0.098	0.131	0.489	0.493
TCRgd %T cell	Pancreatic cancer	Relative count	TBNK	13	0.817(0.684-0.976)	0.026	0	11.342	0.500	0.045	0.040	0.288	0.277
DN (CD4-CD8-) NKT %T cell	Pancreatic cancer	Relative count	TBNK	26	0.862(0.766-0.970)	0.014	9	27.522	0.330	-0.016	0.027	0.556	0.382
CD28+ CD45RA+ CD8dim %T cell	Pancreatic cancer	Relative count	Treg	21	1.162(1.022-1.320)	0.022	0	16.570	0.681	-0.049	0.030	0.119	0.762
CD28+ CD45RA+ CD8dim AC	Pancreatic cancer	Absolute count	Treg	18	1.172(1.013-1.355)	0.033	0	12.244	0.785	-0.045	0.031	0.158	0.748
CD39+ CD8br %T cell	Pancreatic cancer	Relative count	Treg	17	0.808(0.691-0.944)	0.007	37	25.347	0.064	-0.067	0.034	0.067	0.086
CD39+ CD8br %CD8br	Pancreatic cancer	Relative count	Treg	18	0.844(0.720-0.989)	0.036	36	26.760	0.062	0.025	0.038	0.518	0.064
CD39+ CD8br AC	Pancreatic cancer	Absolute count	Treg	19	0.848(0.744-0.967)	0.014	19	22.292	0.219	0.013	0.035	0.709	0.236
CD25++ CD8br AC	Pancreatic cancer	Absolute count	Treg	17	0.852(0.743-0.976)	0.021	0	12.149	0.734	0.039	0.036	0.288	0.776
CD28- CD8br AC	Pancreatic cancer	Absolute count	Treg	11	0.817(0.673-0.991)	0.040	9	10.974	0.360	-0.072	0.064	0.291	0.590
BAFF-R on IgD+ CD38- naive	Pancreatic cancer	MFI	B cell	19	1.101(1.005-1.205)	0.038	0	16.547	0.554	-0.020	0.034	0.566	0.626
CD25 on IgD+ CD24-	Pancreatic cancer	MFI	B cell	15	0.831(0.727-0.951)	0.007	0	11.223	0.668	0.031	0.046	0.516	0.366
CD38 on CD20-	Pancreatic cancer	MFI	B cell	16	1.180(1.002-1.391)	0.048	0	13.702	0.548	-0.056	0.036	0.140	0.584
CD3 on CD28+ CD45RA+ CD8br	Pancreatic cancer	MFI	Treg	19	0.918(0.843-1.000)	0.050	0	14.042	0.726	-0.034	0.023	0.163	0.795
CD34 on HSC	Pancreatic cancer	MFI	Myeloid cell	9	0.906(0.825-0.995)	0.039	0	7.824	0.451	-0.083	0.036	0.057	0.375
HVEM on naive CD8br	Pancreatic cancer	MFI	Maturation stages of T cell	15	1.104(1.014-1.202)	0.022	0	10.447	0.729	0.003	0.032	0.924	0.762
CD25 on CD45RA- CD4 not Treg	Pancreatic cancer	MFI	Treg	16	1.227(1.052-1.432)	0.009	17	18.035	0.261	0.008	0.032	0.804	0.315
CD25 on CD4 Treg	Pancreatic cancer	MFI	Treg	13	1.221(1.013-1.472)	0.036	21	15.183	0.232	0.058	0.038	0.162	0.235
CD25 on CD39+ CD4+	Pancreatic cancer	MFI	Treg	16	1.159(1.019-1.317)	0.025	0	12.152	0.667	0.028	0.031	0.380	0.730
CD64 on CD14+ CD16- monocyte	Pancreatic cancer	MFI	Monocyte	21	0.890(0.801-0.989)	0.030	6	21.169	0.387	0.040	0.025	0.135	0.561
CD64 on CD14- CD16-	Pancreatic cancer	MFI	Monocyte	13	1.187(1.014-1.391)	0.033	0	11.049	0.525	0.011	0.042	0.807	0.533
CD64 on monocyte	Pancreatic cancer	MFI	Monocyte	19	0.830(0.746-0.924)	0.001	0	17.751	0.472	0.022	0.032	0.489	0.605
CD8 on naive CD8br	Pancreatic cancer	MFI	Maturation stages of T cell	12	0.783(0.622-0.987)	0.038	31	15.974	0.142	-0.117	0.045	0.027	0.130
CD4 on CD28+ CD4+	Pancreatic cancer	MFI	Treg	14	1.195(1.025-1.392)	0.023	13	15.022	0.306	0.040	0.045	0.393	0.448
CD4 on CD39+ secreting Treg	Pancreatic cancer	MFI	Treg	14	1.150(1.001-1.321)	0.049	0	6.377	0.931	0.016	0.033	0.643	0.489
CD11b on CD33br HLA-DR+ CD14dim	Pancreatic cancer	MFI	Myeloid cell	12	0.873(0.777-0.982)	0.023	2	11.192	0.427	0.017	0.059	0.776	0.612
HLA-DR on B cell	Pancreatic cancer	MFI	TBNK	11	1.133(1.026-1.252)	0.013	0	4.167	0.940	0.027	0.034	0.449	0.977

MR-PRESSO analysis was not feasible when N_snp ≤ 3.

MR, Mendelian randomization; OR, odds ratio; CI, confidence interval; SE, standard error; MR-PRESSO, Mendelian randomization pleiotropy residual sum and outlier; cDC, conventional dendritic cell; Treg, regulatory T cell; TBNK, T, B, and natural killer cells; MFI, mean fluorescence intensity.

### Exploratory two-step MR analysis of candidate circulating immune mediators

Eight circulating immune phenotypes met the criteria for inclusion as candidate indirect pathways in the analysis of genetically proxied MAPK8 expression and pancreatic cancer risk. These phenotypes included activated and resting CD4+ Tregs, CM DN (CD4−CD8−) %DN, CD8br %T cells, DN (CD4−CD8−) NKT %T cells, CD25++ CD8br AC, CD28− CD8br AC, CD38 on CD20−, and HLA-DR on B cells.

Twelve circulating immune phenotypes were included as candidate indirect pathways in the analysis of genetically proxied *RAF1* expression and pancreatic cancer risk. These phenotypes included activated and resting CD4+ Tregs, CM DN (CD4−CD8−) %DN, TCRγδ %T cells, CD39+ CD8br %T cells, CD39+ CD8br AC, BAFF-R on IgD+ CD38− naive B cells, CD38 on CD20−, CD3 on CD28+ CD45RA+ CD8br cells, CD64 on CD14+ CD16− monocytes, CD64 on CD14− CD16− cells, CD64 on monocytes, and CD8 on naive CD8br cells. Overall, 17 distinct circulating immune phenotypes were included in the two-step MR analyses involving *RAF1* or MAPK8 expression and pancreatic cancer risk (Fig. 2).

**Fig. 2:**
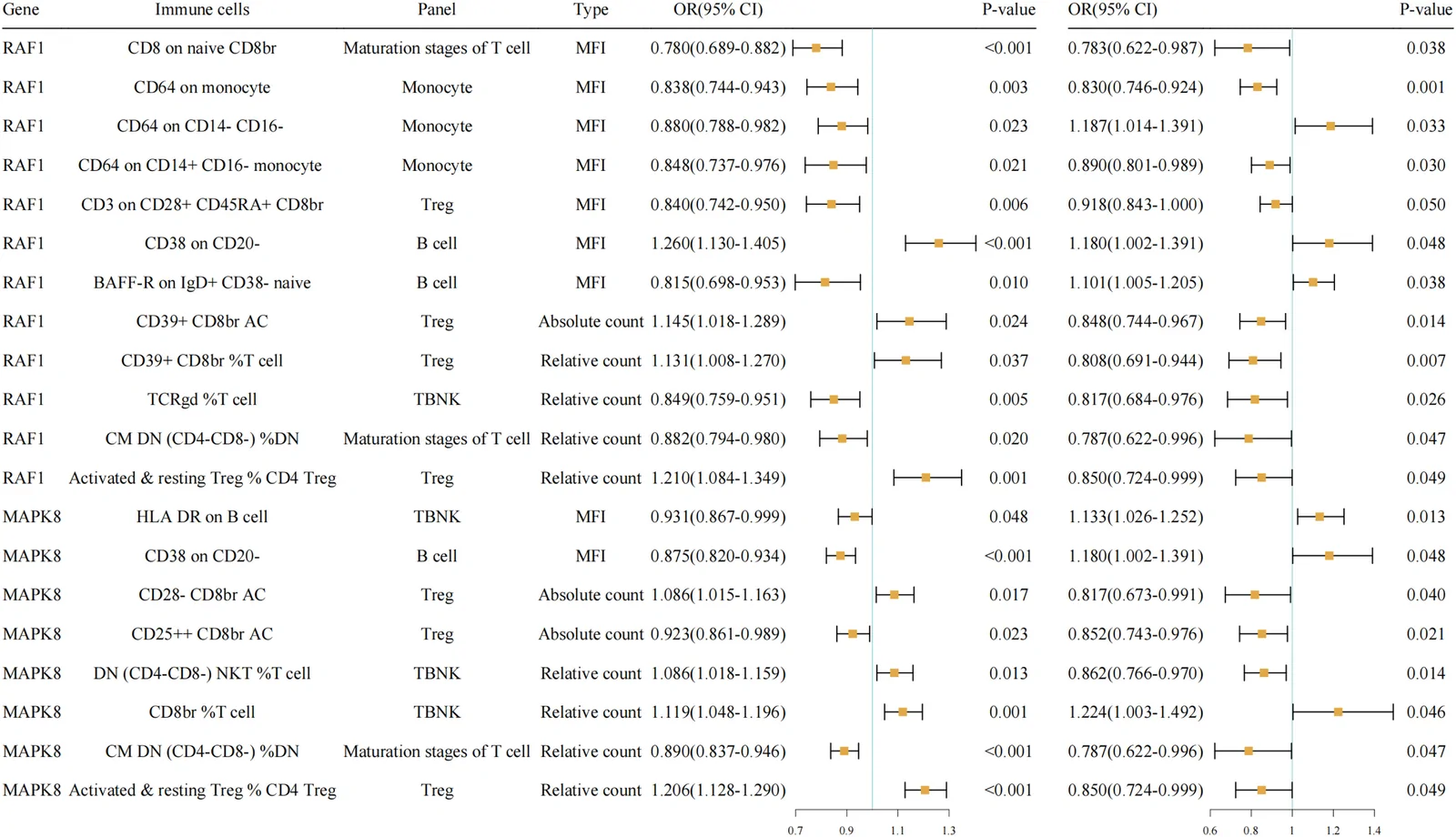
Forest plots of the two-step Mendelian randomization associations for candidate circulating immune phenotypes. The left set of estimates shows the associations of genetically proxied *RAF1* or *MAPK8* expression with each circulating immune phenotype, and the right set shows the associations of the corresponding immune phenotypes with pancreatic cancer risk. Points indicate odds ratios, and horizontal lines indicate 95% confidence intervals.

Among the 17 candidate circulating immune phenotypes, the activated and resting CD4+ Treg-related phenotype was prioritized for detailed interpretation because it showed the largest positive estimated mediation proportion for both MAPK8 and *RAF1*, providing a consistent pattern across the two gene-specific analyses. For MAPK8, the estimated mediation proportion was 16.202% (95% CI: 12.945%–19.460%), with effect estimates of β₁ = 0.187 (P < 0.05), β₂ = −0.162 (P = 0.049), and β₃ = −0.188 (P = 0.001) (Table 4 and Fig. 3a). For *RAF1*, the corresponding estimated mediation proportion was 10.607% (95% CI: 6.950%–14.263%), with effect estimates of β_1_ = 0.190 (P = 0.001), β_2_ = −0.162 (P = 0.049), and β_3_ = −0.291 (P = 0.001) (Table 4 and Fig. 3b).

**Table 4:** Estimated mediation effects of circulating immune phenotypes on the associations of genetically proxied *MAPK8* or *RAF1* expression with pancreatic cancer risk.

Gene	Mediator	β₁	β_2_	Mediating_effect	Direct_effect	Total_effect	Percentage_ mediated (%)
*MAPK8*	Activated & resting Treg % CD4 Treg	0.187	-0.162	-0.03	-0.157	-0.188	16.202(12.945-19.46)
*MAPK8*	CM DN (CD4-CD8-) %DN	-0.116	-0.239	0.028	-0.215	-0.188	-14.823(-17.004–12.643)
*MAPK8*	CD8br %T cell	0.113	0.202	0.023	-0.21	-0.188	-12.132(-14.311–9.954)
*MAPK8*	DN (CD4-CD8-) NKT %T cell	0.082	-0.149	-0.012	-0.175	-0.188	6.534(4.757-8.311)
*MAPK8*	CD25++ CD8br AC	-0.081	-0.161	0.013	-0.201	-0.188	-6.898(-8.707–5.088)
*MAPK8*	CD28- CD8br AC	0.083	-0.202	-0.017	-0.171	-0.188	8.918(7.096-10.739)
*MAPK8*	CD38 on CD20-	-0.133	0.166	-0.022	-0.166	-0.188	11.772(9.319-14.226)
*MAPK8*	HLA-DR on B cell	-0.072	0.125	-0.009	-0.179	-0.188	4.788(3.055-6.521)
*RAF1*	Activated & resting Treg % CD4 Treg	0.19	-0.162	-0.031	-0.26	-0.291	10.607(6.95-14.263)
*RAF1*	CM DN (CD4-CD8-) %DN	-0.125	-0.239	0.03	-0.321	-0.291	-10.29(-13.072–7.508)
*RAF1*	TCRgd %T cell	-0.163	-0.202	0.033	-0.324	-0.291	-11.357(-14.697–8.017)
*RAF1*	CD39+ CD8br %T cell	0.124	-0.213	-0.026	-0.265	-0.291	9.062(6.163-11.961)
*RAF1*	CD39+ CD8br AC	0.136	-0.165	-0.022	-0.269	-0.291	7.682(4.617-10.748)
*RAF1*	BAFF-R on IgD+ CD38- naive	-0.204	0.096	-0.02	-0.271	-0.291	6.719(2.377-11.06)
*RAF1*	CD38 on CD20-	0.231	0.166	0.038	-0.329	-0.291	-13.167(-17.389–8.944)
*RAF1*	CD3 on CD28+ CD45RA+ CD8br	-0.175	-0.085	0.015	-0.306	-0.291	-5.131(-8.736–1.527)
*RAF1*	CD64 on CD14+ CD16- monocyte	-0.165	-0.116	0.019	-0.31	-0.291	-6.609(-10.3–2.918)
*RAF1*	CD64 on CD14- CD16-	-0.128	0.172	-0.022	-0.269	-0.291	7.551(4.678-10.424)
*RAF1*	CD64 on monocyte	-0.177	-0.186	0.033	-0.324	-0.291	-11.321(-14.898–7.744)
*RAF1*	CD8 on naive CD8br	-0.249	-0.244	0.061	-0.352	-0.291	-20.885(-25.484–16.286)

**Fig. 3:**
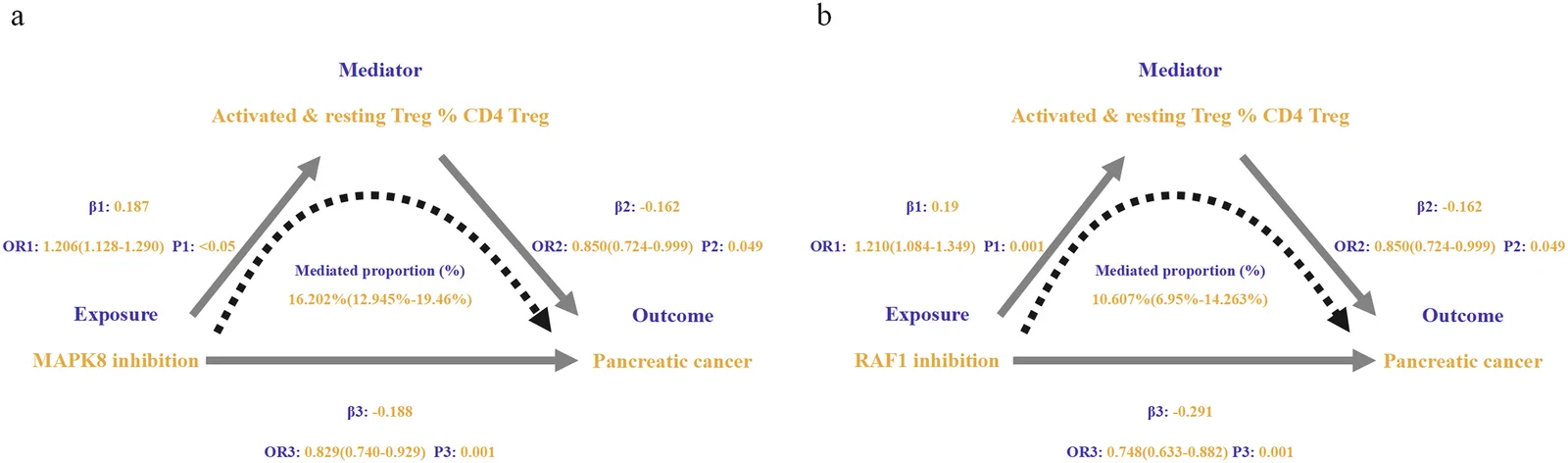
Exploratory two-step Mendelian randomization analysis of the activated and resting CD4+ Treg-related phenotype as a candidate indirect pathway. (a) Associations among genetically proxied *MAPK8* expression, the circulating Treg-related phenotype, and pancreatic cancer risk; (b) associations among genetically proxied *RAF1* expression, the circulating Treg-related phenotype, and pancreatic cancer risk. β_1_ represents the association between genetically proxied gene expression and immune phenotype; β_2_ represents the association between immune phenotype and pancreatic cancer risk; β_3_ represents the total association between genetically proxied gene expression and pancreatic cancer risk. Mediation proportions were calculated based on the product-of-coefficients method.

These estimates suggest that circulating Treg-related immune variation may contribute to a limited part of the associations between *RAF1* or *MAPK8* expression and pancreatic cancer risk. Additional immune and nonimmune pathways may contribute to the remaining effects.

### Associations of genetically proxied *MAPK8* and *RAF1* expression with additional cancer outcomes

As supplementary comparative analyses, we further examined the associations of genetically proxied *RAF1* and *MAPK8* expression with three other cancer outcomes, including non-small cell lung cancer adenocarcinoma, malignant neoplasm of the thyroid gland, and non-Hodgkin lymphoma (Table 5). IVW analysis revealed that *MAPK8* expression was a protective factor against both malignant neoplasm of the thyroid gland (OR = 0.728, 95% CI: 0.634–0.836, *P* < 0.001) and non-Hodgkin lymphoma (OR = 0.825, 95% CI: 0.686–0.993, *P* = 0.042). Similarly, *RAF1* expression was associated with a reduced risk of non-small cell lung cancer adenocarcinoma (OR = 0.641, 95% CI: 0.437–0.941; *P = 0.023*). No heterogeneity was detected between eQTLs for *MAPK8* expression and the two cancers or between *RAF1* expression and non-small cell lung cancer (Supplementary Figs. S9-S11).

**Table 5:** Mendelian randomization estimates for the associations of genetically proxied *RAF1* and *MAPK8* expression with comparison cancer outcomes.

**Exposure**	**Outcome**	**Method**	**Nsnp**	**MR**		**Heterogeneity**			**Horizontal pleiotropy**			**MR-PRESSO**
				**OR (95% CI)**	***P* value**	**I^2^ (%)**	**Cochran's Q**	***P* value**	**Egger intercept**	**SE**	***P* value**	***P* value**
*MAPK8* expression	Malignant neoplasm of thyroid gland	Inverse variance weighted	40	0.728(0.634-0.836)	<0.001	0	32.319	0.767				0.850
*MAPK8* expression	Malignant neoplasm of thyroid gland	MR Egger	40	0.777(0.541-1.117)	0.181	0	32.173	0.735	0.014	0.037	0.705	
*MAPK8* expression	Malignant neoplasm of thyroid gland	Weighted median	40	0.746(0.611-0.910)	0.004							
*MAPK8* expression	Malignant neoplasm of thyroid gland	Simple mode	40	0.777(0.561-1.075)	0.136							
*MAPK8* expression	Malignant neoplasm of thyroid gland	Weighted mode	40	0.747(0.577-0.966)	0.032							
*MAPK8* expression	Other and unspecified types of non-Hodgkin lymphoma	Inverse variance weighted	40	0.825(0.686-0.993)	0.042	0	24.699	0.964				0.969
*MAPK8* expression	Other and unspecified types of non-Hodgkin lymphoma	MR Egger	40	1.075(0.666-1.735)	0.769	0	23.326	0.970	0.057	0.048	0.249	
*MAPK8* expression	Other and unspecified types of non-Hodgkin lymphoma	Weighted median	40	0.762(0.585-0.993)	0.044							
*MAPK8* expression	Other and unspecified types of non-Hodgkin lymphoma	Simple mode	40	0.640(0.416-0.984)	0.049							
*MAPK8* expression	Other and unspecified types of non-Hodgkin lymphoma	Weighted mode	40	0.734(0.533-1.010)	0.065							
*RAF1* expression	Non-small cell lung cancer, adenocarcinoma	Inverse variance weighted	15	0.641(0.437-0.941)	0.023	0	7.430	0.917				0.966
*RAF1* expression	Non-small cell lung cancer, adenocarcinoma	MR Egger	15	0.879(0.257-3.013)	0.841	0	7.150	0.894	0.043	0.082	0.606	
*RAF1* expression	Non-small cell lung cancer, adenocarcinoma	Weighted median	15	0.688(0.415-1.140)	0.147							
*RAF1* expression	Non-small cell lung cancer, adenocarcinoma	Simple mode	15	0.892(0.385-2.070)	0.795							
*RAF1* expression	Non-small cell lung cancer, adenocarcinoma	Weighted mode	15	0.469(0.214-1.025)	0.078							

MR-PRESSO analysis was not feasible when N_snp ≤ 3.

MR, Mendelian randomization; OR, odds ratio; CI, confidence interval; SE, standard error; MR-PRESSO, Mendelian randomization pleiotropy residual sum and outlier.

### PheWAS assessment of broad genetic associations

In a PheWAS evaluating *RAF1* and *MAPK8* genetic instruments across 17,361 binary and 1,419 quantitative traits in the UK Biobank, no associations for either instrument reached the genome-wide significance threshold (*P* > 5 × 10^-8^; Supplementary Fig. S12). The strongest nominal signal was observed for *MAPK8* (lowest *P* = 3.2 × 10^-6^), but this did not meet the multiple-testing threshold. Global pleiotropy tests for *RAF1* and *MAPK8* also revealed nonsignificant differences (*P* = 0.17 and 0.06, respectively). Collectively, no broad genetic pleiotropic associations were detected for *RAF1* or *MAPK8* at the prespecified significance threshold.

## Discussion

In this study, a two-step Mendelian randomization framework was used to examine the associations of genetically proxied *RAF1* and *MAPK8* expression with pancreatic cancer risk and to explore the possible involvement of circulating immune phenotypes. Higher genetically proxied expression of *RAF1* and *MAPK8* was associated with a lower risk of pancreatic cancer. Among the immune phenotypes evaluated, an activated and resting CD4+ regulatory T-cell-related trait showed a possible indirect contribution, with estimated mediation proportions of 16.202% for *MAPK8* and 10.607% for *RAF1*. These observations suggest that systemic immune variation may explain a small proportion of the associations observed for *RAF1* and *MAPK8*.

The MAPK signaling pathway plays a central role in pancreatic tumorigenesis, particularly in tumors harboring oncogenic KRAS alterations. Sustained activation of this pathway promotes tumor-cell proliferation, survival, therapeutic resistance, and interactions between malignant cells and the surrounding stroma [23,24]. *RAF1* is a key component of RAF signaling, while *MAPK8* encodes JNK1, a stress-responsive kinase involved in apoptosis, inflammation, and cellular adaptation. Altered expression or activity of these genes may therefore influence pancreatic cancer through several interconnected biological processes. Studies in colorectal cancer and melanoma have demonstrated the biological and therapeutic relevance of RAF pathway modulation and *RAF1*-related signaling [[25], [26], [27], [28]]. A recent clinical report involving molecularly selected pancreatic adenocarcinoma further supports continued investigation of RAF-directed therapeutic strategies in specific patient subgroups [29].

The exploratory mediation analysis provided additional insight into the potential role of circulating immune regulation. Among the candidate immune phenotypes, the activated and resting CD4+ Treg-related trait was highlighted because it showed the largest positive estimated mediation proportion in both the *MAPK8*- and *RAF1*-related analyses. This finding suggests that circulating Treg-related variation may represent one of several pathways linking *RAF1*- and *MAPK8*-related biology to pancreatic cancer risk. The relatively modest mediation proportions also indicate that a considerable part of the observed associations may involve other mechanisms. These may include additional immune-cell populations, tumor-intrinsic MAPK signaling, stromal remodeling, angiogenesis, metabolic regulation, and inflammatory responses.

Regulatory T cells are essential for maintaining immune tolerance and limiting excessive immune activation. In cancer, however, increased Treg activity may contribute to an immunosuppressive environment and facilitate tumor immune escape. Cytokines and chemokines further influence the balance between immune activation and tolerance within malignant tissues [30,31]. Previous studies in BRAF-altered melanoma have shown that RAF pathway inhibition may be accompanied by changes in tumor antigen expression, T-cell infiltration, and immune signaling [14,32,33]. These observations provide a biological context for potential interactions between MAPK-related pathways and immune regulation.

The immune phenotypes examined in the present study were derived from peripheral blood GWAS data and primarily reflect variation in circulating immune-cell abundance, relative proportions, and surface-marker expression. These measures do not directly assess the localization, infiltration, or functional state of Tregs within pancreatic tumor tissue. The Treg-related finding should therefore be interpreted as evidence of systemic immune variation associated with pancreatic cancer risk. Accordingly, the direction of association observed for circulating Treg-related variation may not directly reflect the biological role of Tregs within pancreatic tumor tissue. Circulating immune phenotypes and tumor-infiltrating immune populations represent related but distinct immunological compartments. It does not provide direct information on immune-cell behavior within the local tumor microenvironment. Circulating Treg-related traits may also capture broader changes in immune homeostasis, inflammatory signaling, and interactions between innate and adaptive immune compartments.

*RAF1* and *MAPK8* are associated with different components of the broader in vitro pharmacological profile of encorafenib. Encorafenib is primarily recognized as a BRAF inhibitor, although in vitro studies have also reported activity against CRAF and binding to JNK1 [17,34]. These observations provide a pharmacological basis for considering *RAF1*- and *MAPK8*-related pathways in the present study. However, the MR analysis reflects genetically proxied differences in gene expression over the life course, whereas pharmacological effects are additionally influenced by dose, treatment duration, tissue exposure, and target selectivity. The present findings therefore provide genetic support for the biological relevance of *RAF1*- and *MAPK8*-related pathways and warrant further pharmacological and functional investigation in pancreatic cancer.

RAF-directed therapies have shown clinical activity in selected BRAF-altered malignancies, particularly when combined with MEK inhibition, EGFR blockade, or immune checkpoint therapy [14,25,26,28,33,35]. These clinical findings support continued investigation of RAF-related pathways in molecularly defined cancers. Their application to pancreatic cancer is likely to depend on tumor genotype, pathway activity, and treatment context. The current results add genetic evidence supporting the biological relevance of *RAF1*- and *MAPK8*-related pathways in pancreatic cancer.

The PheWAS analysis identified no significant associations for the *RAF1* or *MAPK8* genetic instruments across the phenotypes examined. Although *MAPK8* showed the lowest nominal P value, this association did not reach the prespecified multiple-testing threshold, and the global pleiotropy tests were also nonsignificant. Overall, the analysis did not reveal evidence of broad phenotypic effects associated with these instruments. More specific associations may still occur in particular tissues, biological pathways, or disease settings that are not fully represented in the available PheWAS data.

Several strengths of this study merit consideration. The use of genetic instruments reduced the potential influence of conventional confounding and reverse causation. The two-step MR framework also enabled the evaluation of gene-disease associations and candidate immune pathways within a unified analytical design. Multiple MR methods and sensitivity analyses were applied to assess the consistency and robustness of the findings. In addition, the integration of target-related eQTL data, a large pancreatic cancer GWAS dataset, 731 circulating immune phenotypes, and PheWAS information provided a broad basis for investigating the relationships among gene expression, systemic immune variation, and pancreatic cancer risk.

Several limitations should be considered. Genetically proxied expression reflects long-term biological variation and may not fully reproduce the acute, dose-dependent, and tissue-specific effects of pharmacological kinase inhibition. The available eQTL data may also provide limited information on gene regulation within pancreatic tumor cells, stromal cells, and local immune populations. In addition, the immune phenotypes were derived from peripheral blood flow cytometry datasets and therefore mainly represent systemic immune variation, without directly measuring Treg infiltration or functional activity in pancreatic tumor tissue. Given the large number of circulating immune phenotypes examined, the immune-related associations and estimated mediation proportions are best viewed within an exploratory screening framework. The absence of colocalization and pancreatic tissue-specific molecular analyses also limits the interpretation of the gene-level associations. Finally, the genetic datasets included in the present study were predominantly restricted to individuals of European ancestry. Differences in allele frequencies, linkage disequilibrium patterns, and immune-related genetic architecture across ancestral populations may limit the generalizability of the present findings, and validation in more diverse populations will be important in future studies. Future studies incorporating colocalization, tumor-specific eQTL data, single-cell analyses, and functional experiments may help clarify the biological relevance of *RAF1*- and *MAPK8*-related pathways in pancreatic cancer.

## Conclusions

In conclusion, this study provides genetic evidence supporting associations of *RAF1* and *MAPK8* expression with pancreatic cancer risk. The two-step MR analysis further suggested that a circulating activated and resting CD4+ Treg-related phenotype may contribute to a small proportion of these associations, indicating a possible involvement of systemic immune regulation. Further tissue-specific, functional, and pharmacological studies may help clarify the biological relevance of *RAF1*- and *MAPK8*-related pathways in pancreatic cancer and their pharmacological implications in the context of encorafenib.

## Ethics approval and consent to participate

This study utilized publicly available summary-level data from previous genome-wide association studies (GWAS). No new data were collected, and all data were publicly accessible; therefore, no additional ethical approval was required. The original studies that generated these datasets obtained ethical approval from their respective institutional review boards, and all participants provided informed consent. Details of the ethical approvals can be found in the original publications.

## Availability of data and materials

The summary-level genetic datasets analyzed in this study are publicly available through the IEU OpenGWAS Project (https://gwas.mrcieu.ac.uk/). The phenome-wide association study (PheWAS) data were derived from the UK Biobank resource via the AstraZeneca PheWAS portal. All relevant data supporting the findings of this study are included in the article and its supplementary materials. The code used for data analysis in this study can be obtained from the corresponding authors upon reasonable request.

## Funding

This work was supported by the Fujian Provincial Health Technology Project (No. 2021CXA005), the Leading Project Foundation of Science and Technology of Fujian Province (No. 2023Y0053), and the Joint Funds for the Innovation of Science and Technology of Fujian Province, China (No. 2023Y9149).

## CRediT authorship contribution statement

**Fen Lin:** Writing – review & editing, Writing – original draft, Project administration, Methodology, Data curation. **Wenwen Tu:** Writing – review & editing, Writing – original draft, Funding acquisition, Formal analysis. **Ronggui Lin:** Writing – review & editing, Writing – original draft, Data curation. **Han Shi:** Writing – review & editing, Writing – original draft. **Shiyun Lu:** Writing – original draft. **Zongqi Weng:** Writing – review & editing, Writing – original draft, Supervision.

## Declaration of competing interest

The authors declare that they have no known competing financial interests or personal relationships that could have appeared to influence the work reported in this paper.
