# Supplementary material for: Genetic evaluation of encorafenib-associated kinases RAF1 and MAPK8 in pancreatic cancer: Exploratory mediation by circulating immune traits
Source: Transl Oncol. 2026 Sep 22;73:103040. doi: 10.1016/j.tranon.2026.103040 (PMC13633892; doi:10.1016/j.tranon.2026.103040)
Supplement: Supplementary file 1 [file mmc1.docx]

**Supplementary Data**

**Supplementary Table S1.** Pancreatic cancer-associated SNPs identified from RAF1 and MAPK8 eQTL data.

**Supplementary Table S2.** Immune cell-associated SNPs identified from RAF1 and MAPK8 eQTL data.

**Supplementary Table S3.** Pancreatic cancer-associated SNPs identified from immune cell GWAS data.

**Supplementary Table S4.** Positive control-associated SNPs identified from RAF1 and MAPK8 eQTL data.

**Supplementary Table S5.** MR analysis results for causal relationships among RAF1, MAPK8 inhibition, and immune cell subtypes.

**Supplementary Fig. S1.** MR visualization of causal relationships between MAPK8 inhibition and pancreatic cancer. (a) Forest plot of effect estimates across SNPs; (b) Scatter plot of MR methods; (c) Leave-one-out sensitivity analysis; (d) Funnel plot for pleiotropy assessment.

**Supplementary Fig. S2.** MR visualization of causal relationships between RAF1 inhibition and pancreatic cancer. (a) Forest plot; (b) Scatter plot; (c) Leave-one-out analysis; (d) Funnel plot.

**Supplementary Fig. S3.** MR visualization of causal relationships between MAPK8 inhibition and HLA-DR on HLA-DR+ T cells. (a) Forest plot; (b) Scatter plot; (c) Leave-one-out analysis; (d) Funnel plot.

**Supplementary Fig. S4.** MR visualization of causal relationships between MAPK8 inhibition and CD45RA+ CD28- CD8br AC. (a) Forest plot; (b) Scatter plot; (c) Leave-one-out analysis; (d) Funnel plot.

**Supplementary Fig. S5.** MR visualization of causal relationships between RAF1 inhibition and CD45 on CD33- HLA DR- cells. (a) Forest plot; (b) Scatter plot; (c) Leave-one-out analysis; (d) Funnel plot.

**Supplementary Fig. S6**. MR visualization of causal relationships between RAF1 inhibition and CD45RA on naive CD8br cells. (a) Forest plot; (b) Scatter plot; (c) Leave-one-out analysis; (d) Funnel plot.

**Supplementary Fig. S7.** MR visualization of causal relationships between CD86+ plasmacytoid DC AC and pancreatic cancer. (a) Forest plot; (b) Scatter plot; (c) Leave-one-out analysis; (d) Funnel plot.

**Supplementary Fig. S8.** MR visualization of causal relationships between TD CD4+ %T cells and pancreatic cancer. (a) Forest plot; (b) Scatter plot; (c) Leave-one-out analysis; (d) Funnel plot.

**Supplementary Fig. S9.** MR visualization of causal relationships between MAPK8 inhibition and malignant neoplasm of the thyroid gland. (a) Forest plot; (b) Scatter plot; (c) Leave-one-out analysis; (d) Funnel plot.

**Supplementary Fig. S10.** MR visualization of causal relationships between MAPK8 inhibition and non-Hodgkin lymphoma. (a) Forest plot; (b) Scatter plot; (c) Leave-one-out analysis; (d) Funnel plot.

**Supplementary Fig. S11.** MR visualization of causal relationships between RAF1 inhibition and non-small cell lung cancer. (a) Forest plot; (b) Scatter plot; (c) Leave-one-out analysis; (d) Funnel plot.

**Supplementary Fig. S12.** Pan-phenomic association landscape of dual-gene effects: Comparative analysis of MAPK8 and RAF1 across multi-omics domains. (a-b) MAPK8; (c-d) RAF1.
